# Cannabidiol and the Remainder of the Plant Extract Modulate the Effects of Δ9-Tetrahydrocannabinol on Fear Memory Reconsolidation

**DOI:** 10.3389/fnbeh.2019.00174

**Published:** 2019-08-01

**Authors:** Anthony Murkar, Pam Kent, Christian Cayer, Jon James, Tony Durst, Zul Merali

**Affiliations:** ^1^The Royal’s Institute of Mental Health Research affiliated with the University of Ottawa, Ottawa, ON, Canada; ^2^Centre for Advanced Research in Environmental Genomics, Ottawa-Carleton Institute of Biology, University of Ottawa, Ottawa, ON, Canada; ^3^Department of Chemistry and Biomolecular Sciences, University of Ottawa, Ottawa, ON, Canada; ^4^Department of Neuroscience, Faculty of Science, Carleton University, Ottawa, ON, Canada; ^5^Department of Cellular and Molecular Medicine, School of Psychology, University of Ottawa, Ottawa, ON, Canada

**Keywords:** reconsolidation, blockade, fear, memory, cannabinoids, cannabis, THC, CBD

## Abstract

**Background**: Δ9-Tetrahydrocannabinol (THC, a CB1 receptor agonist) and Cannabidiol (CBD, a non-competitive antagonist of endogenous CB1 and CB2 ligands) are two primary components of *Cannabis* species, and may modulate fear learning in mammals. The CB1 receptor is widely distributed throughout the cortex and some limbic regions typically associated with fear learning. Humans with posttraumatic disorder (PTSD) have widespread upregulation of CB1 receptor density and reduced availability of endogenous cannabinoid anandamide, suggesting a role for the endocannabinoid system in PTSD. Pharmacological blockade of memory reconsolidation following recall of a conditioned response modulates the expression of learned fear and may represent a viable target for the development of new treatments for PTSD. In this study, we focused on assessing the impact of the key compounds of the marijuana plant both singly and, more importantly, in concert on attenuation of learned fear. Specifically, we assessed the impact of THC, CBD, and/or the remaining plant materials (post-extraction; background material), on reconsolidation of learned fear.

**Method**: Male Sprague-Dawley rats received six 1.0 mA continuous foot shocks (contextual training). Twenty-four hours later, rats were re-exposed to the context. Immediately following memory retrieval (recall) rats received oral administration of low dose THC, high dose THC, CBD, CBD + low THC, CBD + high THC [as isolated phytochemicals and, in separate experiments, in combination with plant background material (BM)]. Rodents were tested for freezing response context re-exposure at 24 h and 7 days following training.

**Results**: CBD alone, but not THC alone, significantly attenuated fear memory reconsolidation when administered immediately after recall. The effect persisted for at least 7 days. A combination of CBD and THC also attenuated the fear response. Plant BM also significantly attenuated reconsolidation of learned fear both on its own and in combination with THC and CBD. Finally, THC attenuated reconsolidation of learned fear only when co-administered with CBD or plant BM.

**Conclusion**: CBD may provide a novel treatment strategy for targeting fear-memories. Furthermore, plant BM also significantly attenuated the fear response. However, whereas THC alone had no significant effects, its effects were modulated by the addition of other compounds. Future research should investigate some of the other components present in the plant BM (such as terpenes) for their effects alone, or in combination with isolated pure cannabinoids, on fear learning.

## Introduction

Δ9-Tetrahydrocannabinol (THC), the primary psychoactive component of *Cannabis* (*sativa, indica*, and* ruderalis*), has been reported to affect fear memory, expression, consolidation, and extinction (Phan et al., 2008; Lemos et al., 2010; Klumpers F. et al., 2012; Klumpers L. E. et al., 2012; Klumpers et al., 2013; Rabinak et al., 2013, 2014). In addition, Cannabidiol (or CBD), another component of the plant, has also been reported to impact fear memory. Medical cannabis (or marijuana for medical purposes, MMP) is widely used to self-medicate for a variety of medical conditions, including disorders rooted in fear learning such as post-traumatic stress disorder, PTSD (Lucas and Walsh, 2017). However, the effects of marijuana on fear memory reconsolidation have been only sparsely explored. Additionally, MMP is often utilized as a whole plant material. However, the effects of combined doses of THC and CBD in varying concentrations, as well as the role of the remaining (non-THC, non-CBD) plant material and its interactions with THC and CBD, remain largely unexplored. Here, we aimed to identify whether combined THC and CBD could affect fear memory reconsolidation both in isolation and when combined at varying concentrations. In addition, since it is highly relevant for the use of whole plant material as MMP, we also sought to determine whether the effects of THC and CBD on reconsolidation are modulated by the inclusion of the remaining plant material.

Behaviorally, in many respects CBD has been shown to produce effects which are opposite those of THC. One functional magnetic resonance imaging (fMRI) study found CBD and THC had opposite effects on regional activation in the hippocampus, amygdala, superior temporal cortex, and occipital cortex (Bhattacharyya et al., 2010). The same study found that pretreatment with 5 mg CBD intravenously (IV) attenuated the severity of psychotic symptoms induced by THC.

There is some data from central studies to suggest that targeting the endocannabinoid (ECB) system may be a viable strategy for pharmacologically attenuating established fear memories. While THC acts as an agonist for the CB1 and CB2 receptors, both of which have been implicated in fear-learning (Lafenêtre et al., 2007; Ruehle et al., 2012), the actions of CBD are complex. CBD exerts some of its effects indirectly by inhibiting the actions of endogenous CB1 and CB2 agonists. CBD has been shown to act as a potent antagonist for CB1 and CB2 ligands, while displaying low binding affinity for the CB1 receptor (Bisogno et al., 2001; Thomas et al., 2004, 2007). CBD has also been shown to act as an indirect agonist of 5-HT_1A_ receptors, and may exert some of its effects *via* this mechanism (Rock et al., 2012; McPartland et al., 2015). CB1 receptors are expressed in the hippocampus, basolateral and lateral amygdala, and medial prefrontal cortex (mPFC; Tsou et al., 1998)—key regions implicated in fear learning—but are absent in the central and medial nuclei of the amygdala (Katona et al., 2001), regions involved in fear expression. Thus, CB1 likely affects fear expression *via* an indirect neuromodulatory mechanism. CB1 receptors are found on GABAergic neurons of the basolateral amygdala (BLA), and their activation dampens BLA inhibitory interneuron activity. This disinhibition increases output from BLA projections (Marsicano et al., 2002; Pistis et al., 2004). BLA stimulation induces long-term potentiation (LTP) along the BLA-prelimbic (PLC) pathway, and blockade of CB1 transmission prevents this (Tan et al., 2010). The same study also demonstrated that pharmacological blockade of BLA-PLC CB1 signaling blocks encoding of fear learning. Thus there is a strong theoretical framework for the notion that pharmacologically modulating the ECB system may allow for the attenuation of traumatic memories. It is possible that the blockade of CB1 agonists by CBD impedes BLA-PLC signaling, thereby exerting its effects on fear learning.

The distribution of central receptors in the ECB system has already been implicated in human PTSD. Evidence from positron emission tomography (PET) imaging suggests that among those with PTSD there is a widespread upregulation of CB1 receptor density particularly in regions implicated in learned fear (the amygdala, hippocampus, orbitofrontal cortex, and anterior cingulate; Neumeister et al., 2013). Combined with behavioral evidence of cannabinoid involvement in fear-learning, this suggests the endocannabinoid system may be involved in the mediation of fear memories and may represent a viable target for the mitigation of some PTSD symptoms. Indeed, some research points to positive effects of oral THC in the reduction of hyper-arousal and frequency of nightmares among those affected by PTSD (Roitman et al., 2014).

Some findings have also suggested that THC and CBD may disrupt the reconsolidation of recalled fear memories (Lin et al., 2006; Stern et al., 2012), a novel therapeutic strategy that may have relevance for attenuating established memories of trauma. Reconsolidation blockade is the process by which the expression of formed memories is reduced by drugs administered following recall (during the reconsolidation window; Nader et al., 2000; Nader and Hardt, 2009; Pitman et al., 2011). This procedure may offer new avenues for treatment of fear-based disorders that are resistant to extinction. Although several medicinal plants and isolated compounds have been reported to affect fear expression and reconsolidation in rodents (Nader et al., 2000; Lin et al., 2006; Da Silva et al., 2008; Bustos et al., 2009; Stern et al., 2012; Murkar et al., 2016; de Carvalho and Takahashi, 2017), reconsolidation paradigms have had mixed success when translated in human studies (Brunet et al., 2008; Pitman et al., 2011; Spring et al., 2015). Thus, new targets are needed for future translational studies with humans and *Cannabis spp*. extracts may offer one such target.

Although anecdotal findings regarding CBD and THC are compelling, there is a dearth of information around the effectiveness of cannabinoids at blocking fear memory reconsolidation. There is some evidence to suggest that both CBD and THC may block fear memory reconsolidation (Stern et al., 2012, 2015). This is curious, since THC and CBD often produce opposite effects. The effects of combined doses of CBD and THC on fear memory reconsolidation have also been sparsely explored (Stern et al., 2015), albeit at very low doses. It is important to assess this, as the consumption of marijuana would entail exposure to both the main cannabinoids simultaneously. In addition, there may be other active components present in the plant material that may be biologically active and may modulate the effects of the key cannabinoids. However, the effects of other components of the plant in combination with cannabinoids (such as the terpenes) remain largely unexplored. In terms of relevance for human use of MMP, it is important to explore the effects of all components of the plant (since MMP, which is typically administered as whole plant material, does not solely consist of THC and CBD). It is also clear that the concentrations of THC and CBD can vary based on the specific species of the plant; thus it is important to identify and standardize the concentrations of THC and CBD in administered extracts and to verify the effects of the remainder of the plant extracts containing varying concentrations of THC and CBD.

Herein, our experiments examined whether combined doses of THC and CBD would block fear memory reconsolidation, as well as whether the effects of the phytocannabinoids are modulated by the remaining plant background material (BM; all remaining plant components following CBD/THC extraction). In order to simulate MMP preparations consisting of whole plant material (which is much more relevant for human medical cannabis use, which may contain other active non-cannabinoids affecting fear memory), we tested doses of isolated phytochemicals THC and CBD singly, or in combination with each other, and in combination with plant BM. In addition, in order to simulate the effects of varying concentrations of THC in plant material, we tested the effects of co-administration of CBD with both a low- and high-dose of THC (both with and without BM).

## Materials and Methods

### Animals

Male Sprague-Dawley rats (Charles River Laboratories International, Inc., Wilmington, MA, USA; 180–200 g on arrival) were pair housed and maintained on a 12-h light/dark cycle (lights on at 07:00-h). Temperature was maintained at 23°C, and relative humidity at 37%. Throughout the duration of the study, animals had free access to food and water. All experiments were conducted in accordance with the guidelines established by the Canadian Council on Animal Care and approved by the University of Ottawa Animal Care Committee.

### Drugs and Injections

Isolated compounds THC and CBD, as well as plant BM, were extracted from raw plant material of a *Cannabis indica* and *Cannabis sativa* hybrid variety (“Strawberry Kush”). Pure compounds and BM were provided by T. Durst (University of Ottawa, ON, Canada). Plant BM consisted of all other remaining plant components in the extracts following the isolation of THC and CBD. Due to the complexity of completely extracting all the THC and/or CBD, our BM contained less than 3 ± 0.5% THC and less than 0.6% of CBD. Animals were habituated to daily administration of oral almond oil (vehicle; *via* intubation) for 1 week prior to the experiment. In conditions where the BM was co-administered with cannabinoids, the amount of BM was held constant at 30% of the total amount of compounds administered (i.e., treatment dose was 70% cannabinoids and 30% BM; the dose of BM was calculated as $\frac{BM}{\left(THC+CBD+BM\right)}=0.3$). Our low doses of CBD and THC were comparable to moderate doses administered systemically in previous research (Stern et al., 2012, 2015). Stern et al. (2015) observed strong effects on reconsolidation at 10 mg/kg I.P. THC, but did not test at higher doses.

For Experiments 1 and 2, rats were randomly assigned to one of seven treatment groups. (1) 50 mg/kg THC + 21.5 mg/kg BM; (2) 50 mg/kg CBD + 21.5 mg/kg BM; (3) 5 mg/kg THC + 2 mg/kg BM; (4) 50 mg/kg THC + 50 mg/kg CBD + 43 mg/kg BM; (5) 50 mg/kg CBD + 5 mg/kg THC + 24 mg/kg BM; (6) 43 mg/kg BM; and (7) vehicle alone.

For Experiments 3 and 4, in order to explore the effects of isolated cannabinoids in absence of the BM of the plant, rats were randomly assigned to 1 of 4 treatment groups: (1) 5 mg/kg THC; (2) 50 mg/kg CBD; (3) 50 mg/kg THC + 50 mg/kg CBD; (4) 43 mg/kg BM; or (5) Vehicle.

For Experiment 5, rats were similarly randomly assigned to one of five treatment groups: (1) 5 mg/kg THC; (2) 50 mg/kg CBD; (3) 50 mg/kg THC + 50 mg/kg CBD; (4) 43 mg/kg BM; and (5) Vehicle.

### Contextual Fear Conditioning

The conditioning chambers (Coulbourn Instruments) measured 31 cm × 25 cm × 30 cm. The front and back walls were made of clear acrylic, and the two side walls and top made of stainless steel. The floor was composed of 16 stainless steel rods (4 mm diameter spaced 1.4 cm apart) connected to Coulbourn precision regulated animal shockers, which delivered scrambled footshock (1.0 mA). Animals (*N* = 7–10/group) were randomly distributed into treatment groups. Subjects that failed to achieve a minimum baseline freezing level of 40% during re-exposure to the fearful context (memory recall; assessed on Day 2) were removed from the analyses. All experimental procedures were conducted in accordance with methods established by our prior research (Murkar et al., 2018).

### Experimental Procedure

#### Experiment 1: Effects of Plant Extracts With Background Material on Fear Memory Reconsolidation, Short-Term

Animals were exposed to six consecutive 1-s footshocks over the course of 11 min. Contextual conditioning was used (pairing of footshock with the conditioning chamber). Twenty-four hours later, animals were re-exposed to the context in which they received the footshock (conditioning chamber) for a duration of 5 min, and freezing (total time spent in complete immobility) was measured (Day 2; recall). Cage placement and assignment to drug treatment groups were randomized and counterbalanced.

**Figure 1:**
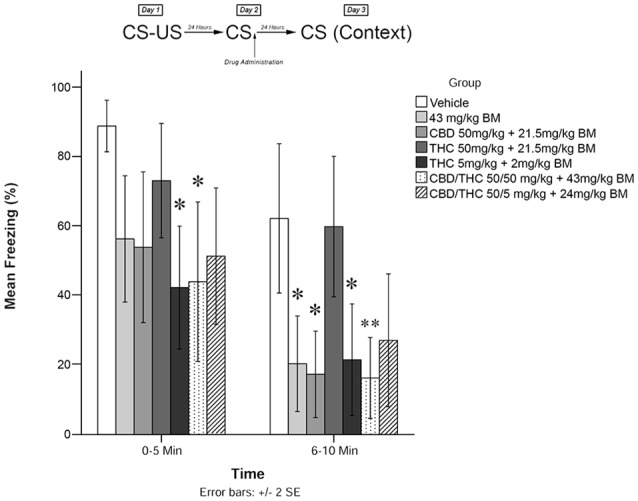
Cannabis extracts with background material (BM) significantly attenuate the reconsolidation of contextual learned fear 24 h after drug administration. *p < 0.05, **p < 0.01.

Immediately following the 5-min recall session, animals were administered drugs according to one of the experimental conditions (low THC + BM; high THC + BM; CBD + BM; high CBD + low THC + BM; high CBD + high THC + BM; BM alone; or vehicle alone). Twenty-four hours later (Day 3; testing), animals were re-exposed to the conditioning chamber and freezing was measured over the course of 10 min. Freezing on Day 3 was scored in two 5-min time blocks (0–5 and 6–10 min). The timeline of procedures used for fear conditioning and the results are illustrated in Figure 1. There were no significant group differences prior to drug administration (see Supplementary Figure S1).

#### Experiment 2: Effects of Plant Extracts With Background Material Reconsolidation of Fear Memory, Long-Term

Using the same training procedures as experiment 1, experiment 2 was conducted to test for long-term effects of isolated cannabinoids in combination with plant BM on fear memory reconsolidation.

**Figure 2:**
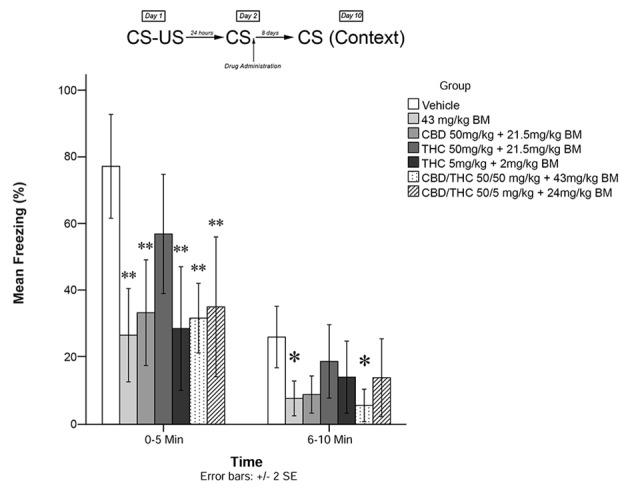
Cannabis extracts with BM significantly attenuated the reconsolidation of contextual learned fear; effect is present on testing day 10. *p < 0.05, **p < 0.01.

Immediately following the 5-min recall session, animals were exposed to one of the experimental conditions (low THC + BM; high THC + BM; CBD + BM; high CBD + low THC + BM; high CBD + high THC + BM; BM alone; or vehicle alone). One week later (Day 10), animals were re-exposed to the conditioning chamber and freezing was measured over the course of 10 min. Freezing on Day 10 was scored in two 5-min time blocks (0–5 and 6–10 min). The timeline of procedures used for fear conditioning and the results are illustrated in Figure 2. There were no significant group differences prior to drug administration (see Supplementary Figure S2).

#### Experiment 3: Effects of Plant Extracts Without Background Material on Fear Memory Reconsolidation, Short-Term

Using the same training procedures as Experiments 1, 3 was conducted to test for short-term effects of isolated cannabinoids in the absence of plant BM, on fear memory reconsolidation.

**Figure 3:**
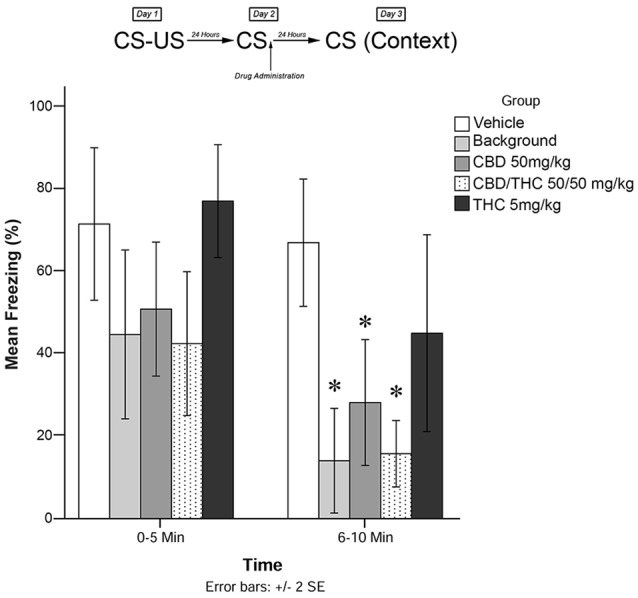
Isolated cannabinoids alone significantly attenuated reconsolidation of contextual learned fear 24 h after drug administration.

Immediately following the 5-min recall session, animals were exposed to one of the following experimental conditions (low THC; high THC; CBD, CBD + THC; BM alone; or vehicle alone). Twenty-four hours later (Day 3; testing), animals were re-exposed to the conditioning chamber and freezing was measured over the course of 10 min. Freezing on Day 3 was scored in two 5-min time blocks (0–5 and 6–10 min). The timeline of procedures used for fear conditioning and the results are illustrated in Figure 3. There were no significant group differences prior to drug administration (see Supplementary Figure S2).

#### Experiment 4: Effects of Plant Extracts Without Background Material on Fear Memory Reconsolidation, Longer-Term

Using the same training procedures as Experiments 1, 4 was conducted to test for longer-term effects of isolated cannabinoids, in the absence of plant BM on fear memory reconsolidation.

**Figure 4:**
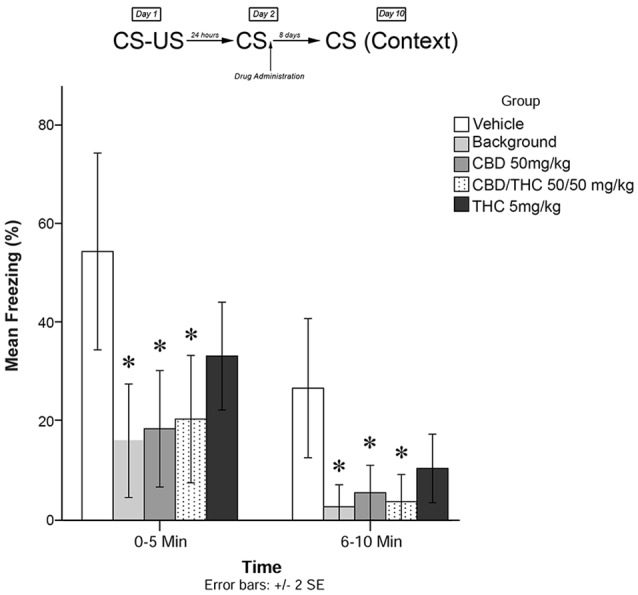
Isolated cannabinoids significantly attenuated the reconsolidation of contextual learned fear; effect is present on testing day 10.

Immediately following the 5-min recall session, animals were exposed to one of the following experimental conditions (low THC; high THC; CBD; CBD + THC; BM alone; or vehicle alone). One week later (Day 10), animals were re-exposed to the conditioning chamber and freezing was measured over the course of 10 min. Freezing on Day 10 was scored in two 5-min time blocks (0–5 and 6–10 min). The timeline of procedures used for fear conditioning and the results are illustrated in Figure 4.

#### Experiment 5: Effects of Plant Extracts in the Absence of Memory Recall (No-Recall Control Conditions)

Experiment 5 was conducted as a control experiment to determine whether blockade of reconsolidation required reactivation of the memory trace. The same training and testing procedures as the other experiments were used, except that recall of the fearful memory trace on Day 2 was absent.

**Figure 5:**
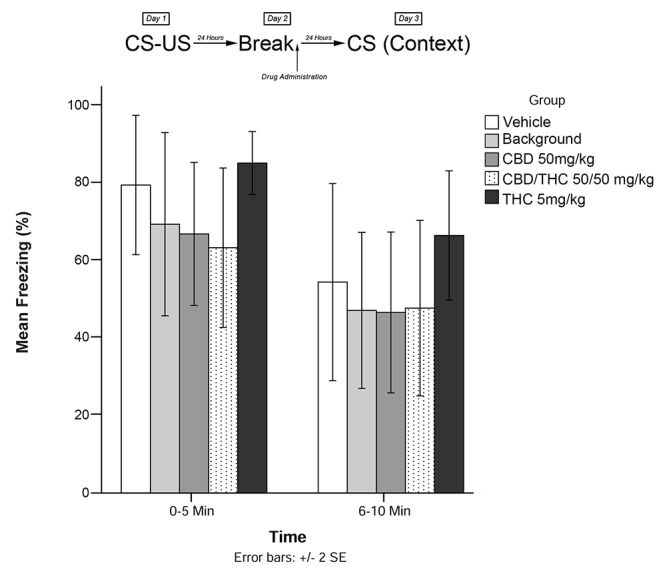
The individual Cannabis extracts on their own had no significant effects on reconsolidation of contextual learned fear 24 h after drug administration in the absence of fearful memory-trace recall.

Animals in this experiment were exposed to one of five treatment conditions (low THC; CBD; CBD + high THC; BM; or vehicle) on Day 2 in home cage (no recall). Animals were then exposed to the conditioning chamber on Day 3, and freezing was measured over the course of 10 min in two 5-min blocks (0–5 and 6–10 min). The timeline of procedures for this no-recall control and the results are illustrated in Figure 5.

### Statistical Analyses

All statistical analyses were conducted using IBM Statistics Package for the Social Sciences^®^ (SPSS) 20. Data were analyzed by mixed-measures analysis of variance (ANOVA), in which drug treatment was the between-groups variable and time was the within-groups variable. Greenhouse-Geisser correction was applied where the assumption of sphericity was violated. Follow-up comparisons of significant main effects and interaction effects were conducted using Bonferroni corrected *t*-tests, or Games-Howell *post hoc* analysis where the assumption of homogeneity of variance was violated.

## Results

### Experiment 1

Figure 1 shows the effects of plant extracts administered immediately post recall on freezing behavior as measured during testing on Day 3. The mixed measures ANOVA revealed a significant main effect of treatment condition on freezing behavior, *F*_(6,53)_ = 5.509, *p* < 0.001.

Follow-up analyses indicated that animals treated with either low THC (5 mg/kg + BM; *p* < 0.05) or CBD + high THC (50 mg/kg each + BM; *p* < 0.05) displayed significantly reduced freezing behavior during the first 5 min of testing. Animals that received low THC (5 mg/kg + BM; *p* < 0.05), CBD (50 mg/kg + BM; *p* < 0.05), or BM alone (*p* < 0.05) following memory recall on Day 2 also displayed significantly less Freezing than vehicle-treated animals during the second 5 min bin of testing on Day 3. Animals that received CBD + high THC (50 mg CBD + 50 mg/kg THC + BM) *p* < 0.01) also displayed significantly reduced freezing on day 3. This suggests that all groups except for high THC + BM (50 mg/kg; *p* > 0.05) and CBD + low THC (50 mg CBD + 5 mg/kg THC + BM, *p* > 0.05) had a significant effect on freezing behavior when co-administered with plant BM.

### Experiment 2

Figure 2 shows the effects of plant extracts administered immediately post recall, on freezing behavior on Day 10 (long-term). The mixed measures ANOVA revealed a significant main effect of treatment group on freezing behavior, *F*_(6,53)_ = 4.974, *p* < 0.001.

Follow-up analyses indicated that animals treated with either low dose of THC (5 mg/kg + BM; *p* < 0.01) or CBD + high THC (50 mg/kg each + BM; *p* < 0.01) displayed significantly reduced freezing behavior during the first 5 min of testing. Animals treated with low dose of THC (5 mg/kg + BM; *p* < 0.01), CBD (50 mg/kg + BM; *p* < 0.01), CBD + low THC (50 mg/kg CBD and 5 mg/kg THC + BM, *p* < 0.01), CBD + high THC (50 mg/kg each + BM, *p* < 0.01), or BM alone (*p* < 0.01) following memory recall on Day 2, displayed significantly less Freezing than vehicle-treated animals during the last 5 min of testing on Day 3. This suggests that all drug treatments except for the high dose of THC + BM (50 mg/kg; *p* > 0.05) had a significant effect on subsequent long-term freezing behavior when co-administered with plant BM.

During the last 5 min of testing, CBD + high THC (50 mg/kg each + BM) yielded significant reductions in freezing behavior (*p* < 0.05), as did BM (*p* < 0.05). All other group effects were non-significant during the last 5 min of testing.

### Experiment 3

Results from Experiment 3 are illustrated in Figure 3. The mixed measures ANOVA revealed a significant main effect of treatment group on freezing behavior, *F*_(4,40)_ = 7.517, *p* < 0.001.

Follow-up analyses indicated that animals that received BM (*p* < 0.05) or CBD + high THC (50 mg/kg each; *p* < 0.05) displayed significantly reduced freezing behavior during the 6–10 min window of testing. Animals that received CBD (50 mg/kg; *p* < 0.05) following memory recall on Day 2 also displayed significantly less Freezing than vehicle-treated animals during the last 5 min of testing on Day 3. This suggests that all drug treatments except for low-dose THC (5 mg/kg; *p* > 0.05) had a significant effect on subsequent freezing behavior when administered as pure compounds (in the absence of plant BM).

### Experiment 4

Figure 4 shows the results of Experiment 4. The mixed measures ANOVA revealed a significant main treatment effect on freezing behavior, *F*_(4,40)_ = 6.670, *p* < 0.001.

Follow-up analyses indicated that animals that received the BM (*p* < 0.05) or CBD + high THC (50 mg/kg each; *p* < 0.05) displayed significantly reduced freezing behavior during the first 5 min and last 5 min of testing on Day 10. Animals that received oral CBD (50 mg/kg) following memory recall on Day 2 also displayed significantly less Freezing than control (vehicle-treated) animals during both the first 5 min (*p* < 0.05) and last 5 min (*p* < 0.05) of testing on Day 10. This suggests that all drug treatments except for low-dose THC (5 mg/kg; *p* > 0.05) had a significant effect on subsequent longer-term freezing behavior when administered as isolated compounds without plant BM.

During the last 5 min of testing, CBD + high THC (50 mg/kg each) yielded significant reductions in freezing behavior (*p* < 0.05), as did BM (*p* < 0.05). All other group effects were non-significant during the last 5 min of testing.

### Experiment 5

Figure 5 shows the results of Experiment 5. Analyses revealed no significant main effects of group for Experiment 5, *F*_(4,40)_ = 0.919, *p* > 0.05.

## Discussion

Our findings suggest that CBD can modulate reconsolidation of learned fear, potentially opening up new treatment avenues for fear-based disorders. Our results also demonstrated that THC at the doses used (the primary psychoactive component of the plant) had no discernible effects on its own, but when co-administered with CBD and/or whole plant BM, was effective in modulating the response (suggesting the effects of THC on fear learning are influenced by other components of the plant). Our studies also revealed an inverted “u” dose response, such that at low- and high-dose THC had opposite effects when co-administered with plant BM. Low-dose THC, but not high-dose THC, attenuated reconsolidation of learned fear, when co-administered with BM. However, these effects were dependent on mediation by co-administration of either CBD or BM. In contrast to previous work (Stern et al., 2015), we found no significant effects of pure THC on reconsolidation of contextual learned fear. We also observed no attenuation of the learned fear expression, when drugs were administered without recall (re-exposure to the conditioned stimulus), suggesting the effect was dependent upon recall of the fearful memory.

These findings partially support prior work which suggests the effects of THC are synergized by the addition of other compounds (either in combination with CBD or whole plant material; Carlini et al., 1974; Fairbairn and Pickens, 1981; Mechoulam and Ben-Shabat, 1999). Research suggests that the behavioral effects of THC are modified by co-administration of other compounds, increasing some potentially therapeutic effects while diminishing sedative and anxiogenic effects (Russo, 2011), and our findings would seem to partially confirm this. However, since BM significantly attenuated the reconsolidation of learned fear on its own, it is unclear whether this is a synergistic effect of THC administered with whole plant material. Since the effects of 5 mg/kg THC were not augmented by co-administration (and since the effect of BM persisted when administered without the addition of THC), it is possible that therapeutic effects resulted primarily from the BM alone rather than THC-BM synergism.

The effects of cannabinoids on fear learning might also be mediated by other factors. CB1 receptors have been shown to play a role in modulating the release of other neurotransmitters such as acetylcholine and dopamine (Piomelli, 2003; Terzian et al., 2011; Micale et al., 2013). Knock-out mice lacking CB1 receptors on dopamine expressing neurons (type-1 receptors; D1Rs) have enhanced expression of cued fear (Terzian et al., 2011), and mice lacking CB1 receptors on neurons expressing D1Rs also exhibited deficits in safety learning in a step-down avoidance task in one study (Micale et al., 2017). Similarly, the effects of CB1 activation on anxiety-like behavior in rodents is partially dependent upon GABAergic and glutamatergic factors (Rey et al., 2012). It is therefore likely that the neuromodulatory effects of CB1 activation on a variety of neurochemical networks play a complex role in the effects of cannabinoids on fear learning as well.

Since BM was essentially devoid of THC and CBD but contained all remaining plant components, a number of molecules could potentially have had effects on reconsolidation of learned fear on their own. Furthermore, THC and CBD precursors cannabidiolic acid (CBDA) and tetrahydrocannabinolic acid (THCA) can be decarboxylated to CBD and THC (Marks et al., 2009), and were present in the BM in small quantities, and could potentially be transformed to active cannabinoid molecules THC and CBD over time. The quantity of THC in our BM sample was 3 ± 0.5% and a minute quantity of CBD (less than 0.3%). The resulting dose of active THC may have been sub-anxiolytic on its own; however, it is likely that the effects of low-dose THC in the BM could be potentiated by some of the other molecules in the BM (e.g., terpenoids). In such a case, we could anticipate a potentiation of the behavioral effects of 5 mg/kg THC by the BM when co-administered. In our experiments this was not the case, but a floor effect due to very low freezing levels among both conditions (BM alone and 5 mg/kg THC plus BM) may have masked such an effect.

Another possibility is that other non-THC, non-CBD constituents of the plant modulated fear learning on their own. *Cannabis spp*. BM contains a number of terpenoid molecules, some of which have been shown to affect anxiety and fear learning. β-Caryophyllene (BCP), for example, is present in *Cannabis spp*. and has been shown to exert anxiolytic-like activity in rodents (Bahi et al., 2014). Anxiolytic effects of BCP are blocked by CB2 antagonist AM630 (Bahi et al., 2014), but not by 5-HT_1A_ antagonist NAN-190 or the GABA_A_ Benzodiazepine partial agonist Flumazenil (Galdino et al., 2012). This suggests BCP may act through CB2 receptors to produce anxiolytic-like effects in rodents. However, the exact mechanism by which BCP exerts its effects remains unknown. In addition, the terpenoids present may vary significantly among different plant strains (in our case, a detailed analysis of the terpenoids present in the BM is not available). Clearly, further research is needed in this regard. Our experiments are also not without limitations. Here, we did not conduct an in-depth dose-response of BM. Furthermore, our animals were pair-housed which could potentially result in the social transmission of fear, and be a confounding factor. In our experience, however, the stress of isolation through single housing is potentially a more severe confounding factor. Last but not least, one might highlight the potential confounding effects of locomotor effects of THC on subsequent freezing as a limiting factor. However, the elimination of THC in the rat is biphasic, with an initial rapid drop in THC levels in the first 120 min post-administration followed by a slower elimination half-life of THC afterward (Klausner and Dingell, 1971; Tseng et al., 2004). 11-hydroxy- Δ9-THC (the primary active metabolite of THC) is rapidly eliminated, and levels of 11-OH- Δ9-THC return to baseline only 240 min post-administration in both male and female rats (Tseng et al., 2004). As a result, the behavioral effects of THC in the rat are short-lived. This is reflected in our data, since our THC 50 mg/kg group exhibited freezing levels that were nearly identical to those of the vehicle-treated animals, 24 h later.

In order to simulate MMP, dosages of BM in our studies were maintained at a constant 30% of total compounds administered for each group. Future studies should aim to conduct dose-response experiments with BM alone and in combination with THC and CBD. Studies should also aim to explore the effects of isolated terpenes (*Cannabis*-derived) alone and in combination with THC and/or CBD. Finally, future studies could benefit from central microinjection studies aimed at sites known to play a role in fear learning (e.g., BLA, CA1, mPFC), and attempt to block the effects with co-administration of antagonists, to help identify the potential locus (loci) of action.

With regards to humans, behavioral studies suggest that the response to marijuana in individuals self-medicating for PTSD varies with symptom type and severity. Wilkinson et al. (2015) for example, found that symptom severity and violent behavior are significantly worse among veterans with PTSD who self-medicated with marijuana. It may be the case that those individuals with greater symptom severity were more likely to seek out alternate means to self-medicate. Indeed, veterans with PTSD are more likely to use marijuana and synthetic cannabis products than veterans without PTSD (Grant et al., 2016). Further evidence suggests that individuals with PTSD with marijuana dependence have blunted emotional reactivity, supporting the notion that cannabinoids may affect fear expression (Tull et al., 2016). Our results suggest that by using reconsolidation paradigms, prolonged treatment (or chronic use) may not be necessary in order to alleviate learned fear. Also, since CBD by itself was effective in our study, administration of CBD alone (i.e., a non-psychoactive component) may potentially be effective without exposing individuals to long-term treatment with psychoactive substances.

While these insights are valuable, the bulk of recent research on the effects of cannabinoids in humans with PTSD has observed the effects through the lens of self-medication (rather than clinical trial), and the lack of experimental guidance of drug administration in studies utilizing raw plant material leaves open the question of whether differences in plant composition may have led to variability in results (and whether different components of the plants—e.g., THC, CBD, etc.—are present in differing ratios and hence differentially affect PTSD symptomology). Since our findings revealed an inverted-u shaped dose response for THC in combination with BM and CBD, it is important that (as we have done here) studies using plant-derived cannabinoids characterize the specific THC and CBD content of extracts and raw plant material. Our studies also demonstrated that the plant BM is not necessarily inert, and also exerts effects on reconsolidation of fear memory. This suggests THC and CBD are not the only fear memory modulating molecules contained within the plant. Although CBD and THC (when co-administered with other compounds) may modulate fear learning, future research should be cautious to identify and quantify the THC, CBD, and other compounds that may be contributing to the measured effects on behavior.

Karniol et al. examined the effects of CBD alone as early as 1974 (Karniol et al., 1974), and found that oral CBD reduced THC-induced anxiety. The same group later demonstrated that CBD could block the effects of THC in normal, healthy participants (Zuardi et al., 1982). More recently, synthetic cannabinoid Nabilone has been demonstrated to effectively reduce the frequency of nightmares in sufferers of PTSD (Fraser, 2009; Cameron et al., 2014). Interestingly, oral THC has been shown to reduce amygdala activation in response to images of threat-related faces (Ballard et al., 2012); however, this contradicts earlier findings showing THC alone may be anxiogenic (Karniol et al., 1974). As a result, it is clear that our current understanding of the role of the endocannabinoid system in PTSD, anxiety, and learned fear is far from complete. Our findings seem to suggest that—at least at the doses used—THC by itself is not sufficient to modulate fear learning, but needs to be co-administered either with CBD, or with other compounds in the cannabis plant, to be effective. Since evidence suggests that PTSD is characterized by upregulation of CB1 receptors and reduced availability of anandamide, it may be the case that CB1 receptor activation by pharmacologic agents might serve to compensate for this receptor upregulation and restore the normal “tone” of the endocannabinoid system over time. However, effects of acute vs. chronic activation by pharmacological agents may not necessarily be the same. Future research should, therefore, aim to clarify the acute effects of CB1 agonists vs. chronic use, as well as examining differences in effects among normal, healthy subjects vs. those at risk of having altered endocannabinoid activity (e.g., sufferers of PTSD).

## Conclusion

Both past and recent data cumulatively support the notion that CBD may impart anxiolytic action (Blessing et al., 2015). In addition, the action of THC in animal and human models of fear-learning warrants further clinical research to elucidate whether cannabinoids may serve as a novel intervention(s) for fear-related disorders such as PTSD. It will be important for these trials to identify the other potentially non-psychoactive components of *Cannabis spp*. to determine if and how they mediate fear learning. It goes without saying that ongoing and future studies aimed at unraveling the mechanism(s) of action of THC and CBD are critically important to fully exploit the therapeutic potential of these pharmacologic targets. Finally, it would be interesting to better understand if and how various pharmacologically active components of *Cannabis spp*. may interact to modulate the signaling of relevant brain circuits (e.g., pathways linking cortex and the amygdala) to affect encoding, consolidation, and reconsolidation of learned fear.

## Ethics Statement

All experiments were conducted in accordance with the guidelines established by the Canadian Council on Animal Care and approved by the University of Ottawa Animal Care Committee.

## Author Contributions

AM wrote the article, analyzed data, performed experiments, and contributed to study design. JJ and CC performed experiments. ZM and PK contributed to study design and revised the article. TD provided the plant extracts and pure compounds used in the experiments.

## Conflict of Interest Statement

The authors declare that the research was conducted in the absence of any commercial or financial relationships that could be construed as a potential conflict of interest.
