# Supplementary material for: Cannabidiol and the Remainder of the Plant Extract Modulate the Effects of Δ9-Tetrahydrocannabinol on Fear Memory Reconsolidation
Source: Front Behav Neurosci. 2019 Aug 1;13:174. doi: 10.3389/fnbeh.2019.00174 (PMC6686031; doi:10.3389/fnbeh.2019.00174)
Supplement: Supplementary file 1 [file Data_Sheet_1.docx]

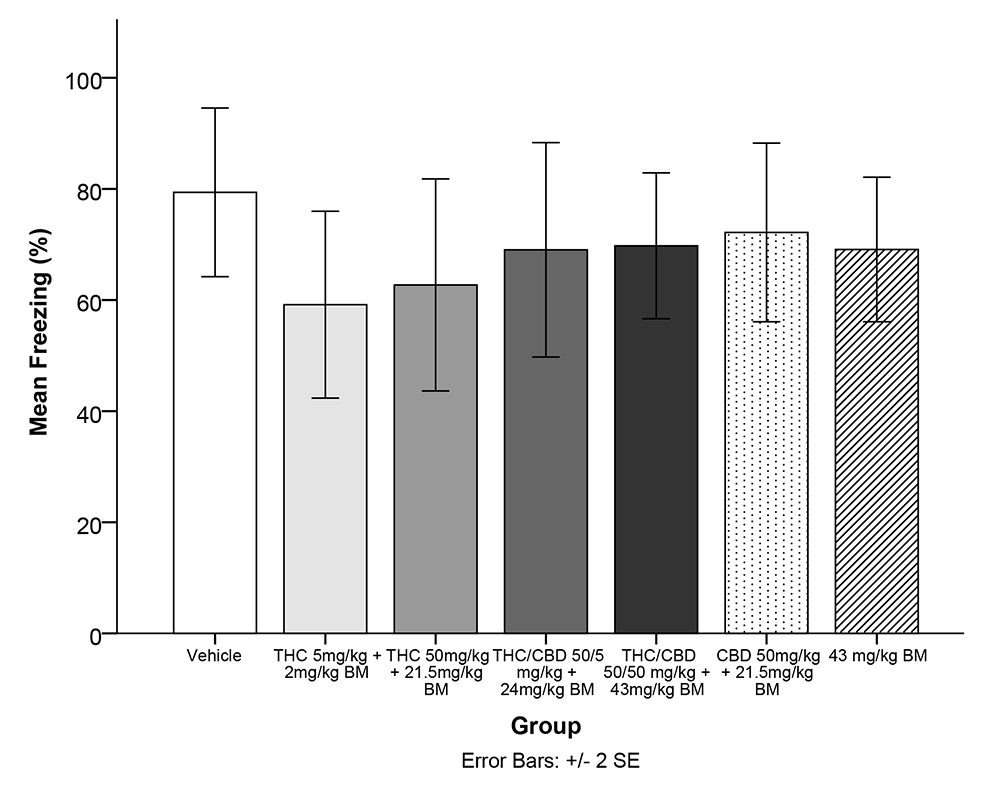


**Supplementary figure 1**. The results of a one-way ANOVA comparing freezing for the drug groups during the brief recall session prior to drug administration on day 2 (experiments 1 and 2) showed no significant differences between the groups. Levene’s test indicated that the assumption of homogeneity of variance was not violated, *p* > 0.05. Analyses revealed no significant main effects of group, F(6,53) = 0.649, *p* > 0.05.


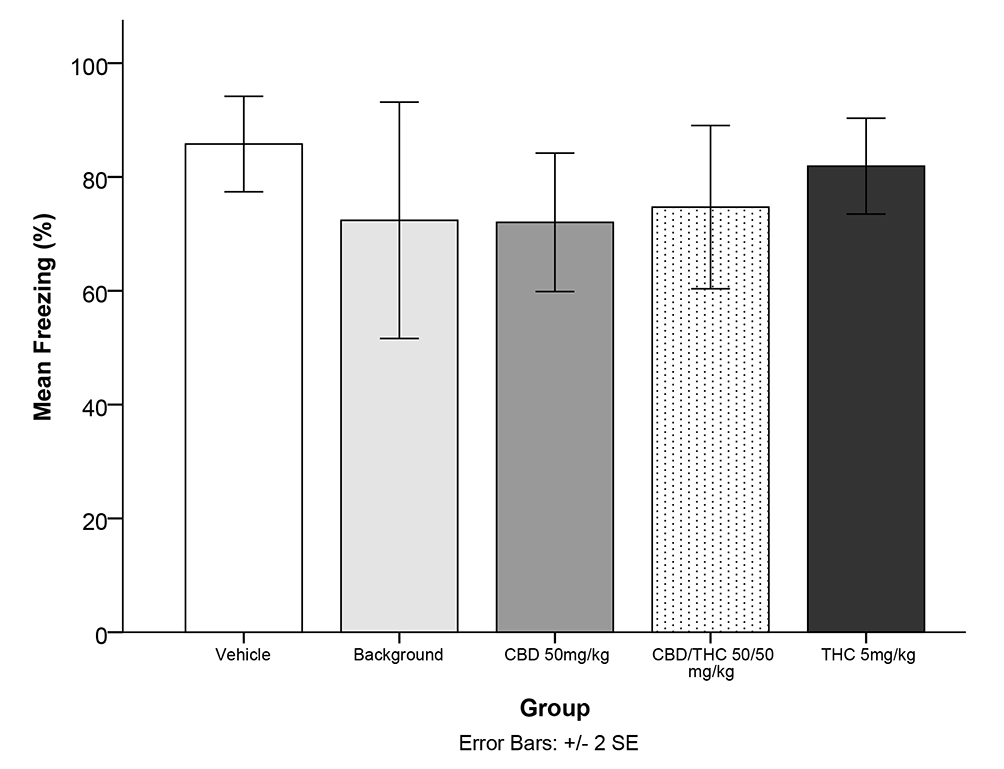


**Supplementary figure 2**. The results of a one-way ANOVA comparing freezing for the drug groups during the brief recall session prior to drug administration on day 2 (experiments 3 and 4) showed no significant differences between the groups. Levene’s test indicated that the assumption of homogeneity of variance was not violated, *p* > 0.05. Analyses revealed no significant main effects of group, F(4,44) = 0.819, *p* > 0.05.
